# Suppressors of RNAi from plant viruses are subject to episodic positive selection

**DOI:** 10.1098/rspb.2013.0965

**Published:** 2013-08-22

**Authors:** Gemma G. R. Murray, Sergei L. Kosakovsky Pond, Darren J. Obbard

**Affiliations:** 1 Centre for Infection Immunity and Evolution, Institute of Evolutionary Biology, University of Edinburgh, Edinburgh, UK; 2 Department of Genetics, University of Cambridge, Cambridge, UK; 3 Department of Medicine, University of California San Diego, San Diego, CA, USA

**Keywords:** molecular evolution, positive selection, evolutionary arms race, RNA interference, viral suppressor of RNAi, RNA silencing suppressors

## Abstract

Viral suppressors of RNAi (VSRs) are proteins that actively inhibit the antiviral RNA interference (RNAi) immune response, providing an immune evasion route for viruses. It has been hypothesized that VSRs are engaged in a molecular ‘arms race’ with RNAi pathway genes. Two lines of evidence support this. First, VSRs from plant viruses display high sequence diversity, and are frequently gained and lost over evolutionary time scales. Second, *Drosophila* antiviral RNAi genes show high rates of adaptive evolution. Here, we investigate whether VSRs diversify faster than other genes and, if so, whether this is a result of positive selection, as might be expected in an arms race. By analysis of 12 plant RNA viruses, we show that the relative rate of protein evolution is higher for VSRs than for other genes, but that this is not attributable to pervasive positive selection. We argue that, because evolutionary time scales are extremely different for viruses and eukaryotes, it is improbable that viral adaptation (as measured by the ratio of non-synonymous to synonymous change) will be dominated by one-to-one coevolution with eukaryotes. Instead, for plant virus VSRs, we find strong evidence of episodic selection—diversifying selection that acts on a subset of lineages—which might be attributable to frequent shifts between different host genotypes or species.

## 1. Introduction

The interests of viruses and hosts often conflict: for a virus, host infection is necessary for replication, whereas for a host, infection can cause disease. This relationship exerts selective pressures on both host and virus, which may result in reciprocal adaptation and counter-adaptation in the form of an evolutionary ‘arms race’ [1]. At the genetic level, such arms races have been described for host and virus proteins that directly interact, and particularly in those involved in host antiviral immunity and viral evasion of host immunity [2–4]. The interaction between the RNA interference (RNAi) antiviral immune system of many eukaryotes and viral suppressors of RNAi appears to have the potential to instigate such an arms race [5,6].

RNAi-related pathways perform a range of functions in eukaryotes, but common to all is the role of short RNA molecules (approx. 20–30 nucleotides) in recognizing and manipulating complementary nucleotide sequences [7,8]. These systems have been found across eukaryotes [9], and function as an antiviral immune system in many lineages, including plants [10], *Drosophila* [11], mosquitoes [12], nematode worms [13] and fungi [14]. Antiviral RNAi pathways involve the Dicer family (Dcr) of proteins, which are members of the Ribonuclease III family of enzymes, the Argonaute family (Ago) [15], and various accessory proteins. Briefly, the pathway involves the recognition of viral dsRNA by Dcr, which dices it into short interfering RNAs (siRNAs). These are loaded into an Ago-containing effector complex, where one siRNA strand is lost and the other used to target and cleave RNA with the complementary sequence [7]. In plants [16] and in some animals [17], the small RNA signal is amplified and exported, resulting in non-cell-autonomous antiviral defence.

Many viruses express products that actively block the function of the antiviral RNAi pathway, termed viral suppressors of RNAi (VSRs), or RNA silencing suppressors (RSSs) [7,18]. VSRs are thought to be ubiquitous in viral genera. They have been found in RNA and DNA viruses, with both plant and animal hosts [18]. Suppression of the antiviral RNAi pathway by a VSR may often be a key stage of viral infection [7], and some viruses even encode multiple VSRs (e.g. potyviruses; P1 and HcPro) [19,20]. VSRs may inhibit the viRNAi pathway at various stages. Some bind dsRNA and sequester siRNAs away from the RNAi pathway. These include P10 of vitiviruses [21], NS3 of tenuiviruses [22], the NSs of tosposviruses [23], and the joint function of HcPro and P1 from potyviruses [24]. The 2b protein of cucumoviruses binds to Ago, preventing the RNA-induced silencing complex (RISC) from cleaving target RNA [25]. The P0 of poleroviruses induces the degradation of Ago [26]. Others inhibit cell-to-cell signalling of immunity, for example, the P30 of tobamoviruses [27] and 16k protein of tobraviruses [28]. A number of VSRs interfere with the pathway in multiple ways. For instance, HcPro inhibits both immunity in the infected cell and cell-to-cell signalling [29], and the P25 of potexviruses has been found to both prevent long-distance signalling [30] and induce the degradation of Ago [31]. On the other hand, it has been observed that the P1 of sobemoviruses inhibits the viRNAi pathway in the infected cell by removing siRNAs from the cell, but enhances the signalling of cell-to-cell immunity [32].

If the genes mediating antiviral RNAi pathways were engaged in a classical one-to-one arms race with VSRs, both host and virus genes might be expected to undergo rapid diversifying evolution under the force of strong positive selection. Consistent with this scenario, three key proteins in the antiviral RNAi pathway of *Drosophila* (Dcr-2, Ago-2 and R2D2) are among the most rapidly evolving genes in the *Drosophila* genome, and population-genetic analysis suggests that this is due to positive selection rather than relaxed constraint [33,34]. In addition, signatures of recent and recurrent selective sweeps can be found in Ago2 and Dcr2 across many *Drosophila* species [35,36].

If the genes controlling antiviral pathways are evolving rapidly and adaptively as the consequence of arms race selection, then VSRs are good candidates for the source of the antagonistic selection that drives this. There is some anecdotal evidence of rapid evolution in VSRs in viruses that infect plants. First, VSRs found in different viral families have no detectable sequence homology, even when their functions are similar, suggesting rapid evolution or multiple independent acquisitions [7,37]. Second, some VSRs appear to have arisen recently, perhaps as the result of adaptation to a host, suggesting the existence of selective pressure on VSR function [18]. Third, some VSRs in plant viruses show high protein sequence diversity within viral species relative to other genes (e.g. HcPro in potyviruses [20]), which is consistent with rapid evolution.

If the VSRs of plant viruses were engaged in an arms race with their host, this might be detectable as an elevated rate of non-synonymous substitutions (d*N*) relative to the rate of synonymous substitutions (d*S*), and thus a higher d*N*/d*S* ratio for VSRs than for other viral genes. However, while an elevated d*N*/d*S* might be suggestive of adaptive arms-race-driven evolution, it may also result from relaxed constraint. To test specifically for adaptive evolution one can compare the model fit for models of sequence evolution in which some codons evolve adaptively (d*N*/d*S* > 1) with those in which all codons are constrained to evolve neutrally or under selective constraint (d*N*/d*S* ≤ 1) [38,39].

While a conventional arms race scenario implies constant reciprocal adaptation in both host and virus, in reality viral host-shifts can be frequent relative to the time scale of host evolution, so that selective pressures on the virus may vary across viral lineages. Therefore, in addition to testing for pervasive positive (diversifying) selection, we also took advantage of recent advances in the modelling of sequence evolution to test for episodic diversifying selection.

We performed these tests on all the known coding regions of the genomes of 12 plant viruses with described VSRs. These were selected because they have well-characterized VRSs and substantial publicly available genetic data. We compared the rates of protein evolution of VSRs with other genes and found that although VSRs did show elevated rates of non-synonymous to synonymous substitution, there was no evidence of ubiquitous positive selection, as might have been expected from a simplistic one-to-one arms race. Instead, we found strong evidence of episodic adaptation, consistent with coevolutionary dynamics that involve strong, but intermittent, positive selection.

## 2. Material and methods

### (a) Sequence data

We searched the literature for publicly available data from single-stranded RNA viruses of plants with known VSRs. We chose not to include animal viruses as there are relatively few with well-characterized VSRs, and none of these has substantial population-genetic data. We identified 41 such viruses (see electronic supplementary material, table S1), but 29 of these had fewer than five alignable non-identical isolates in GenBank, making them unsuitable for phylogenetic analysis of adaptive sequence evolution because of the low power of such analyses on small alignments [40]. Our dataset, therefore, comprised the remaining 12 viruses, spanning 10 distinct viral genera (table 1). Some include more than one ‘named’ viral taxon, although all are predominantly from the species named, and all have divergence in a suitable range for our analyses. We have chosen to treat the P1 protein of potyviruses as a VSR, as it enhances the VSR activity of HcPro and, in the absence of HcPro, has evolved to act as a suppressor in its own right [46]. The datasets contained an average of 57 non-identical isolates (range 5–100). The within-species diversity varied substantially between genes and viruses: the average tree length for the viruses was 3.5 expected substitutions per codon (range 0.5–11.9), average gene length was 486 codons (range 17–2920), and average non-recombinant gene segment length was 336 codons (range 9–1711; see electronic supplementary material, table S2). Coding sequences for each viral gene were aligned using ClustalW in Bioedit [47] and adjusted by eye (alignments are available in the electronic supplementary material).

**Table 1.** Viruses analysed, their VSRs and which part of the viRNAi pathway they are thought to target.

family, genus	species	VSR	function	references
Alphaflexiviridae, *Potexvirus* (ssRNA+)	potato virus X (PVX)	P25	Argonaute and signal	[30,31]
Bunyaviridae, *Tospovirus* (ssRNA−)	tomato-spotted wilt virus (TSWV)	NSs	siRNA	[23]
Betaflexiviridae, *Vitivirus* (ssRNA+)	grapevine virus A (GVA)	P10	siRNA	[21]
Bromoviridae, *Cucumovirus* (ssRNA+)	cucumber mosaic virus (CMV)	2b	Argonaute	[41,42]
Luteoviridae, *Polerovirus* (ssRNA+)	sugarcane yellow leaf virus (SYLV)	P0	Argonaute	[43]
Potyviridae, *Potyvirus* (ssRNA+)	turnip mosaic virus (TurMV)	P1 and HcPro	siRNA and signal	[24,44,45]
	plum pox virus (PPV)			
	potato virus Y (PVY)			
Sobemovirus, *Sobemovirus* (ssRNA+)	rice yellow mottle virus (RYMV)	P1	siRNA	[32]
Tenuivirus, *Tenuivirus* (ssRNA+)	rice stripe virus (RSV)	NS3	siRNA	[22]
Unknown, *Tobamovirus* (ssRNA+)	tobacco mosaic virus (ToMV)	P30	signal	[27]
Unknown, *Tobravirus* (ssRNA+)	tobacco rattle virus (TRV)	16K	signal	[28]

### (b) Recombination and phylogenetic reconstruction

Since recombination can mislead phylogenetic analyses [48], we tested each gene alignment for evidence of recombinants using the GARD analysis implemented in datamonkey.org [49]. Genes in which recombination was detected were divided at the inferred break-points prior to the construction of phylogenetic trees [48]. For phylogenetic analysis by maximum likelihood (PAML) [39], trees were constructed for each non-recombinant gene segment by MrBayes [50], using a partitioned (site-specific) rate model in which each codon position is ascribed a different rate. Run length ranged from 10 000 to 500 000 MCMC iterations, and chain convergence was determined by comparing two parallel runs and ensuring that variance in split frequencies dropped below 0.05. Burn-in length was determined by visual inspection of changes in log-likelihood over the MCMC, and maximum clade-credibility trees were used in downstream analysis. For the analysis using HyPhy [38], trees were constructed for each gene as part of the GARD analysis after using a codon model selector to determine the optimal model.

### (c) Analysis of sequence evolution

Estimates of relative rates of protein evolution and tests for positively selected sites and classes of site (i.e. with d*N* > d*S*) were obtained through a phylogenetic approach implemented with two software packages: Paml v. 4 and HyPhy. An ‘evolutionary fingerprint’ analysis, which quantifies the pattern of constant positive selection and constraint across codons, a clustering analysis on these ‘fingerprints’ and a test for episodic selection were applied with HyPhy only, since comparable tests were unavailable in Paml [51,52]. Where possible, both packages were used to guard against our results being an artefact of a particular methodology or a set of assumptions.

*Codeml* (PAML) fits a codon substitution model to an alignment conditional on a phylogenetic tree using maximum likelihood [53]. *Codeml* model M0 was used to estimate a single best-fit d*N*/d*S* (ω) for each non-recombining gene segment, with confidence intervals calculated by the curvature method implemented in *codeml* [54], and two pairs of other models (*codeml* models M1a versus M2a and M8a versus M8) were used to test for site-specific positive selection through likelihood ratio tests (LRTs) [39]. In addition to fitting the rate class parameters to the data, *codeml* implements a ‘Bayes empirical Bayes’ approach that infers posterior probabilities of site classes for each site [55]. These probabilities were used to identify sites under positive selection.

The HyPhy package [38] provides three different pre-configured tests for the detection of site-specific positive selection, all of which were applied to the data: SLAC (single likelihood ancestor counting), REL (random effects likelihood) and FEL (fixed effects likelihood). REL was also used to provide an estimate of the average relative rate of protein evolution for each gene, expressed as d*N* − d*S* to avoid numerical issues when d*S* is zero, which is possible because HyPhy permits synonymous substitution rates to vary from site to site, while PAML fixes d*S* across sites [56]. PARRIS (a PARtitioning approach for Robust Inference of Selection), which allows site-variable d*S*, was used to provide a comparison with the LRTs in *codeml*. These HyPhy analyses were performed using the online interface www.datamonkey.org [57].

We also performed the ‘evolutionary fingerprinting’ and clustering analysis in HyPhy, which fits a general discrete bivariate model of evolutionary rates across a gene [51], with the number of rate classes in the model determined by the data. The ‘evolutionary fingerprint’ describes the joint distribution of synonymous and non-synonymous rates across codons within each gene, and the similarity between two fingerprints is quantified by a distance metric (termed the evolutionary selection distance, ESD). Calculating a distance matrix for a set of genes, allows us to compare their fingerprints.

Finally, we applied a recently developed mixed-effects model of evolution (MEME) test for site-specific episodic selection in HyPhy [52]. In general, tests for positive selection are relatively insensitive to brief periods of selection, as subsequent constraints can obscure a brief elevation in dN. However, MEME tests whether a non-zero proportion of branches is evolving with d*N* > d*S* at each site, thereby gaining power to detect selection. The key difference between MEME and other methods is that the former require the *mean* d*N*/d*S* at a site to be greater than 1 when averaged over time (termed ‘pervasive’ or ‘ubiquitous’ positive selection), while MEME also detects bursts of selection followed by conservation that often yield mean d*N*/d*S* < 1, which would be missed by conventional approaches (termed ‘episodic’ positive selection). Simulation suggests that MEME is considerably more powerful than the other approaches, but equally accurate, often discovering 3–4 times the number of sites subject to episodic selection than are subject to pervasive selection [52].

### (d) Statistical analysis of d*N*/d*S* and d*N*−d*S*

A meta-analysis of the gene-wise d*N*/d*S* estimates was performed to test for a difference between VSRs and other genes. We applied a variance (assuming variance∼mean) stabilizing transformation (log) to the d*N*/d*S* point estimates. The delta method was applied to determine the variances of the log-transformed estimates [58]. We failed to normalize the distribution of average d*N*−d*S* for each gene, and therefore only non-parametric methods were used on this measure. A model of the transformed point estimates of d*N*/d*S* estimates was fitted using the restricted maximum-likelihood software package ASReml [59]. In the model, gene class (i.e. VSR or non-VSR) was treated as a fixed effect, and gene and viral family were treated as random effects. Numerical variance estimates obtained from PAML were taken into account by weighting the estimates inversely by the transformed variances.

Two non-parametric tests were also applied to test for a difference in d*N*/d*S* (or d*N*−d*S*) between VSRs and other genes. A Mann–Whitney *U*-test was performed on both the d*N*/d*S* and d*N*−d*S* estimates, though this test fails to account for the effect of virus species on the rate of evolution. In addition, the probability of the observed rankings of VSRs (when ordered by d*N*/d*S* or d*N* – d*S*) within each virus was calculated through use of Fisher's method of combining the *p*-values for each individual virus (i.e. the probability that a VSR has the observed rank or higher, given the number of genes in that virus). This test treats d*N*/d*S* (or d*N*−d*S*) as a factor nested within species.

### (e) Statistical analysis of tests for site-specific selection

Few positively selected sites were discovered using the tests for pervasive positive selection. However, such tests may suffer from deficiencies in power. Despite this, if VSRs are under an unusually strong selective pressure, which may be expected under the reciprocal selection (arms race) scenario, we might expect them to be more frequently identified as containing a class of positively selected sites than other types of genes. This hypothesis was tested using Fisher's exact tests (FETs) on the numbers of VSRs and non-VSR genes where positively selected sites were or were not detected. To evaluate potential bias in these tests, the statistics that jointly determine their power were also tested, and no significant difference was found between VSRs and other genes through Mann–Whitney *U*-tests on sample size (*p* = 0.22), tree length (*p* = 0.21) and gene length (*p* = 0.60).

Many positively selected sites were discovered using the MEME test for episodic selection. Therefore, for this analysis, we performed tests on the proportion of sites detected by MEME at *p* ≤ 0.05 as having a non-zero fraction of branches with d*N* > d*S*. We calculated the probability of the cumulative observed ranking of VSRs when ordered by proportion of branches under episodic selection (as done with the d*N*/d*S* and d*N*−d*S* estimates) and performed a Mann–Whitney *U*-test on the proportions of sites detected. Despite the increased sensitivity of MEME compared with other approaches, power is finite and there will be unknown false-negatives.

### (f) Statistical analysis of evolutionary fingerprint analysis

The significance of VSR clustering in the evolutionary fingerprint analysis was tested using a permutation test, allowing the comparison of the null distribution of ESDs between VSRs (estimated by permuting distances to calculate a null distribution) and the observed average ESD between VSRs to be compared.

## 3. Results

### (a) Mean d*N*/d*S* is higher for viral suppressors of RNAi than for other genes

**Figure 1.**
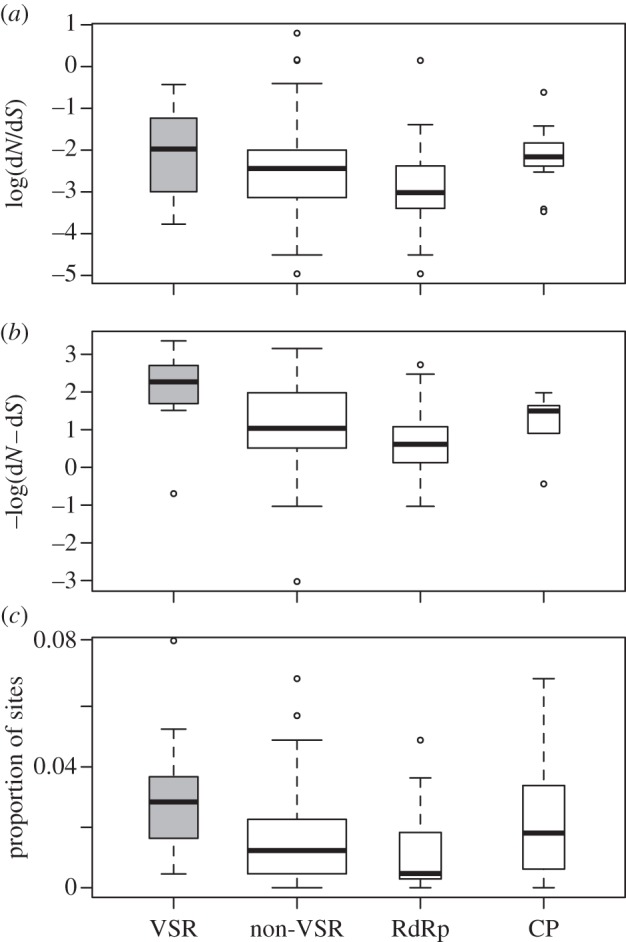
Boxplots of (*a*) log(d*N*/d*S*) estimates from PAML, (*b*) −log(d*N*−d*S*) estimates from REL and (*c*) MEME estimates of the proportion of sites under episodic selection. (*a*), (*b*) and (*c*) categorize these estimates into VSR genes (grey) and all other (non-VSR) genes, including coat proteins and RNA-polymerases, and also show the coat protein (CP) and RNA-dependent RNA polymerase (RdRp) genes separately. Widths of boxes reflect number of genes.

The meta-analysis suggests that VSRs evolve with a significantly higher mean d*N*/d*S* ratio than other classes of viral genes (Wald test: *p* < 0.001; see figure 1*a,b*; electronic supplementary material, table S3), although the effect is small (d*N*/d*S* effect size = 0.04). This was true whether or not the relative rate of protein evolution was modelled as a function of viral species. This result was supported by a statistically significant Mann–Whitney *U*-test performed on the d*N*−d*S* estimates from REL (HyPhy; *p* = 0.044), although not by the d*N*/d*S* estimates from *codeml* (PAML; *p* = 0.089). In addition, the VSR has the highest average d*N*/d*S* of any gene in six out of the 12 viruses we tested using the *codeml* (PAML) estimates and five out of 12 using the REL (HyPhy) estimates. The high ranking of the VSRs is unlikely to be by chance (PAML d*N*/d*S* ranking *p* = 0.025 and REL d*N*−d*S* ranking *p* = 0.011, using Fisher's method for combining *p*-values).

However, it is known that certain types of viral genes are subject to significantly higher constraint than others. Therefore, the observation that VSRs evolve faster than other viral genes might not result from positive selection on VSRs, but rather from reduced constraint relative to other gene families. Tests for positive selection are required to determine this.

### (b) Viral suppressors of RNAi do not show evidence of ubiquitous diversifying selection

LRTs for site-specific positive selection acting on VSRs did not provide evidence of consistent positive selection across VSRs (table 2; electronic supplementary material, table S4). Moreover, it was found that VSRs are no more likely to test positive than non-VSRs (PAML: *p* = 0.54 and PARRIS: *p* = 0.57, FET; but note that the power to detect selection will differ between genes). Similarly, site-specific analyses using REL (HyPhy) and *codeml* M8 (PAML) neither consistently detected positively selected codons in VSRs nor detected them significantly more frequently in VSRs than in other genes (FET, REL: *p* = 1, M8: *p* = 1, SLAC: *p* = 0.21, FEL: *p* = 1; table 3; electronic supplementary material, table S4). The results from these methods were not consistent, but this is not surprising; the tests use different criteria and vary in power and accuracy in detecting different patterns of selection in different datasets. Nevertheless, no VSR shows consistent positive results across all tests.

**Table 2.** Number of genes within a gene class (VSR; coat protein, CP; RNA-polymerase, RdRp; other; and non-VSR total) that showed significant evidence of positive selection (*p* < 0.05) and numbers of genes that did not (*p* > 0.05) through LRTs in PAML (M8a versus M8) and PARRIS (HyPhy).

gene class	PAML (M8a versus M8)		PARRIS	
	*p* < 0.05	*p* > 0.05	*p* < 0.05	*p* > 0.05
VSR	5	10	1	14
CP	6	6	0	12
RdRp	1	10	0	11
other	10	33	3	40
non-VSR total	17	49	3	63

**Table 3.** Numbers of genes within a gene class (VSR; coat protein, CP; RNA-polymerase, RdRp; other; and non-VSR total) that did and did not have sites that were inferred to be evolving under positive selection (with *ω* > 1) by REL (HyPhy), M8 (codeml PAML), SLAC (HyPhy) and FEL (HyPhy).

gene class	HyPhy REL		PAML M8		HyPhy SLAC		HyPhy FEL	
sites with *ω* > 1	present	not present	present	not present	present	not present	present	not present
VSR	9	6	5	10	7	8	10	5
CP	10	2	7	5	5	7	9	3
RdRp	5	6	4	7	3	8	9	2
other	25	16	12	29	10	31	23	18
non-VSR total	40	24	23	41	18	46	41	23

### (c) Viral suppressors of RNAi evolutionary fingerprints do not cluster together

**Figure 2.**
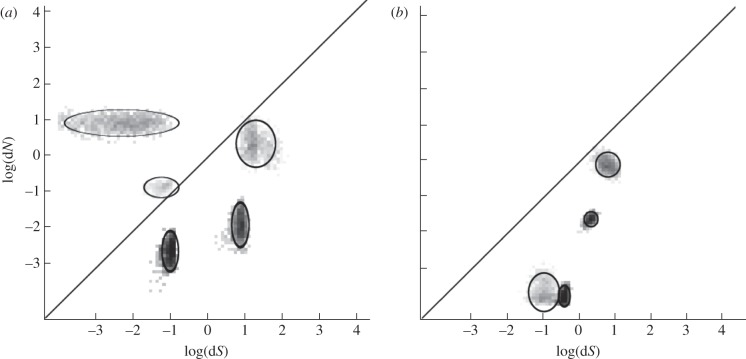
VSR fingerprints for (*a*) sugarcane yellow leaf VSR and (*b*) tobacco mosaic VSR. These plots describe the rate classes that have been inferred from the data: log(d*N*) against log(d*S*). The depth of colour represents the weight of a given estimate of the point *ω* value for that rate class. The ellipses are centred on approximate sampling means. The diagonal line represents a neutral rate (d*N* = d*S*). Rate classes evolving under positive selection are above the line, and ones evolving under constraint are below the line.

**Figure 3.**
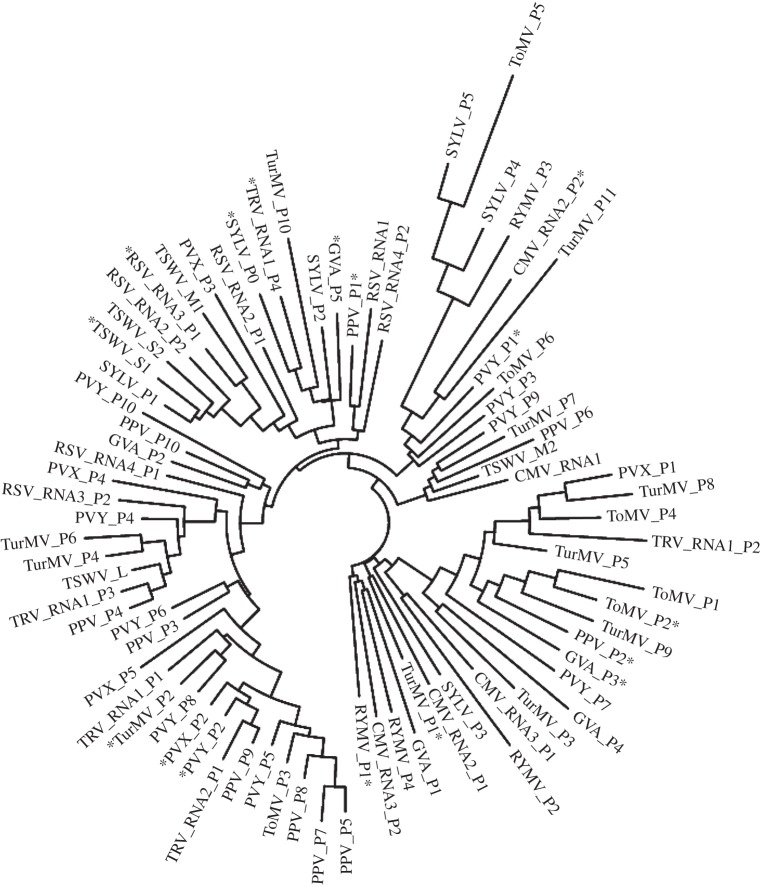
Clustering diagram of evolutionary distances between fingerprints. VSRs are asterisked. The length of the branches indicates the evolutionary selection distance (ESD) between genes.

Evolutionary ‘fingerprints’ (the bivariate discrete distributions of d*N*/d*S*) were found to be no more similar between VSRs than would be expected by chance (*p* = 0.31 by permutation test), indicating that pervasive selective pressures shaping their evolution do not set them apart from other viral genes (example fingerprints in figure 2; all given in electronic supplementary material, figure S1; clustering diagram in figure 3). The same was found for coat proteins (*p* = 0.14) and polymerases (*p* = 0.21), which have been identified in almost all of the viruses in our dataset. Consistent with the tests we performed for pervasive positive selection, VSR fingerprints do not consistently have a class of sites with ω > 1.

### (d) Viral suppressors of RNAi are subject to unusually extensive episodic selection

In seven of the 12 viruses tested, a VSR is the gene with the greatest proportion of sites evolving under episodic selection as identified by MEME (see electronic supplementary material, table S4). The *p*-value for the likelihood of the observed ranking of VSRs within viruses under the null of random rankings (with respect to VSR) is 0.0008 (using Fisher's method for combining *p*-values). Under a Mann–Whitney *U*-test of the ranking of the VSRs with respect to other genes (not accounting for variation between viruses) it is found that VSRs have higher proportions of sites under episodic selection than other genes (*p* = 0.024; figure 1*c*). Furthermore, the pattern we observed in mean d*N*/d*S* and d*N*−d*S* across different types of genes (VSRs, non-VSRs, RdRps and CPs) is broadly reflected in the patterns in the proportion of sites found to be evolving under episodic selection (figure 1).

## 4. Discussion

We were unable to identify a ubiquitous elevated rate of adaptive evolution in VSRs when compared with other genes, as might have been expected under a conventional one-to-one arms race scenario. Although VSRs did show significantly higher mean rates of protein evolution (quantified in different analyses by d*N*/d*S* and d*N*−d*S*), few of them showed significant evidence of ubiquitous adaptive evolution, and this was not significantly different to the rate of ‘positive’ tests for genes with other functions. Thus, the slightly elevated rate of protein evolution in VSRs might be due to reduced constraint compared with other genes, such as polymerases, which are known to be highly conserved. However, VSRs do display a strikingly high proportion of sites evolving under episodic selection as identified by MEME. While MEME is capable of detecting both episodic and pervasive selection, the test detected a much larger number of sites under selection than tests that are insensitive to episodic selection (see electronic supplementary material, table S4); thus we conclude that episodic, rather than pervasive selection, is the driving force behind the MEME results.

Below, we suggest that the null result for conventional one-to-one arms race selection is unlikely to be due to low power, and that it instead reflects a potential asymmetry in selective responses between the host and the virus. This asymmetry means that, while host evolution can certainly drive adaptive substitutions in the virus (and vice versa), it is unlikely to result in a significantly elevated d*N*/d*S* ratio in the virus. Additionally, the high frequency of episodic selection in VSRs may suggest that the dominant positive selective pressures on viruses, at least over observable time scales, results from variation between host immune systems (genotype-to-genotype or species-to-species) rather than host adaptation.

### (a) Power to detect selection is high

The power and accuracy of the tests for positively selected sites are dependent on sample size, tree length, gene length, and the pattern and strength of selection. Although the inferences that can be drawn from power comparisons with simulated datasets are limited, such studies suggest our power to detect moderate-to-strong persistent selection should have been substantial. For example, Wong *et al*. [60] tested simulated data consisting of sequences of 500 codons with a tree length of three and 30 isolates. They found that when 10 per cent of sites are evolving with *ω* = 5, 45 per cent with *ω* = 1 and 45 per cent with *ω* = 0, an LRT results in 76 per cent true-positives and no false-positives. Similarly, simulations by Kosakovsky Pond *et al*. [38] using 250 codons, tree length 3 and 32 isolates suggest that the HyPhy REL analysis should provide a very powerful and moderately accurate test when one-fifth of sites are under positive selection with *ω* between 2 and 4 (nominal Bayes factor of 50; REL yields approx. 95% true-positives and 20% false-positives). In our dataset, the mean sample size was 57 sequences and the mean total tree length was 3.5 substitutions per codon; average gene length was 486 codons (see electronic supplementary material, table S2). These results suggest that our *codeml* (PAML) M8a/M8 and REL analyses should provide substantial power to test for strong selection, and provide a less powerful but valid test for weak selection. Thus, it seems unlikely that the apparent lack of strong selection acting on VSRs reflects low power alone.

### (b) Ubiquitous positive selection acts only on a subset of viral suppressors of RNAi

We find that some VSRs show evidence of persistent positive selection, but that others do not, and this may be a true reflection of the evolutionary process. Even though VSRs as a group do not show evidence of pervasive diversifying selection, it is possible that the participation in an arms race is not uniform across VSRs, but rather that some VSRs are responding to selective pressures by rapid evolution and others are not. For example, in our analyses, 2b (CMV) shows evidence of adaptive evolution in all but the PARRIS analysis, which appears to be a conservative test, while NSs (TSWV) and P0 (SYLV) both had positive results in all but two tests. Nevertheless, we were unable to identify any mechanistic basis for differences in rate. VSRs can be categorized by how they suppress RNAi. NSs (TSWV), P10 (GVA), P1 (RYMV), NS3 (RSV), and HcPro and P1 (potyviruses) are thought to inhibit the accumulation of siRNAs; 2b (CMV), P0 (SYLV) and P25 (PVX) are thought to act on Argonaute; and P25 (PVX), HcPro and P1 (potyviruses), P30 (ToMV) and 16K (TRV) are thought to inhibit cell-to-cell signalling. However, none of these groups consistently showed evidence of persistent positive selection across different analyses.

Host range is a further factor that could influence patterns of pervasive positive selection across viral species. Host range data on 11 of the viruses was downloaded from the Plant Viruses Online database [61] (see electronic supplementary material, table S5). The detection of positive selection in VSRs, the rate of protein evolution across all genes and the proportion of sites detected to be under episodic selection were found to be uncorrelated with any measure of host range—namely, number of known susceptible species (*S*_s_), number of known susceptible families (*F*_s_), proportion of tested species susceptible (*S*_t_) and proportion of tested families susceptible (*F*_t_). However, among VSRs, the rate of protein evolution (rather than the probability of testing ‘positive’) and the proportion of sites found to be under episodic selection appear to be weakly correlated with some measures of host range. Specifically, d*N*/d*S* was positively correlated with *S*_s_ (linear model, no correction for multiple testing, *p* = 0.001; Spearman's rank correlation coefficient, *r*_s_ = 0.37), *F*_s_ (*p* = 0.018; *r*_s_ = 0.35), *S*_t_ (*p* = 0.016; *r*_s_ = 0.55), but not *F*_t_ (*p* = 0.25 *r*_s_ = 0.38). Similarly, the proportion of sites under episodic selection correlated with some measures of host range (*p* = 0.001, *r*_s_ = 0.49 for *S*_s_; *p* = 0.033, *r*_s_ = 0.44 for *F*_s,_; *p* = 0.13, *r*_s_ = 0.42 for *S*_t_; *p* = 0.70 , *r*_s_ = 0.19 for *F*_t_). This may suggest that host range plays a role in determining the strength or frequency of episodic selection on VSRs. However, experimental host range may be poorly known in many groups, and may not reflect host range in the wild. For this dataset, while *S*_s_ and *F*_s_ are correlated (*p* = 0.00014), and *S*_t_ and *F*_t_ are correlated (*p* = 0.0010), *S*_s_ and *F*_s_ do not correlate with *S*_t_ and *F*_t_ (for species: *p* = 0.18; for families: *p* = 0.40). This suggests that sampling strategies may have varied across viruses, and thus that this dataset is not ideal for such an analysis. This therefore warrants further study when the data allow.

### (c) The separation of evolutionary time scales may make reciprocal coevolution hard to detect

Even if an arms race does occur, and it is reciprocal in the sense that adaptation in each party selects for counter-adaptation in the other, the elevated rate of adaptive evolution might be undetectable in the virus because of the different time scales over which evolution occurs in eukaryotes and viruses. Imagine a hypothetical scenario in which every amino acid substitution across the host genome was driven by one-to-one reciprocal coevolution with a single virus, and every amino acid substitution across the whole viral genome was similarly driven by one-to-one reciprocal coevolution with that host. Data from *Drosophila* suggest this may be on the order of one adaptive amino acid substitution every 50 years [62] for a multicellular eukaryotic host with large effective population size, short generation time and relatively compact genome, and it is unlikely to be substantially higher for most plants [63]. By the assumption of one-to-one reciprocity, this would drive one adaptive amino acid fixation every 50 years in the virus. Assuming synonymous substitutions are neutral, given eukaryotic mutation rates on the order of 1 × 10^–8^ site^−1^ yr^−1^ and viral mutation rates of 1 × 10^−3^ site^−1^ yr^−1^, and respective genome sizes of 15 000 two-kb protein-coding genes and 10 one-kb protein-coding genes, this would amount to genome-wide d*N*/d*S* ∼ 0.1 for the host, but a d*N*/d*S* that was 50–100-fold lower for the virus. Even more extreme scenarios, such as a 1 : 10 host : virus ratio of substitution, would still be difficult to detect, and the presumption that every single host substitution (regardless of gene) would mediate novel selection of the virus is unrealistically favourable to the detection of selection. Thus, the high mutation rate in RNA viruses may make such coevolutionary selection very hard to detect. This is in sharp contrast to the rapid adaptive evolution seen in viruses that infect vertebrates, whose evolution is not primarily driven by coevolution with the host, but by antagonism with an acquired host response that adapts plastically over the same time scale that governs viral evolution [64,65].

### (d) Episodic selection and a one-sided arms race

There are at least two other reasons why we might not observe ubiquitous positive selection in these viruses. First, it has been suggested [4] that, while capable of rapid change, viruses are also under very high constraint owing to the necessity of successful interaction with the host for replication [66]. Second, viruses are able to move between hosts and host populations. High constraint may make it easier for a virus to move between hosts with varying immune systems than to adapt to a particular host immune system [4]. If this is the case, the selective pressure that drives evolution in RNAi genes might not result from rapid evolutionary change within VSRs, but result instead from changes in the composition of the viral community infecting particular host species. If the viruses that move between host species have sufficiently divergent VSRs, this process could also drive rapid evolution in the host. Although unknown, it seems plausible that this viral community changes rapidly over evolutionary time, given the rate of evolution in vertically transmitted genomic parasites such as transposable elements [67,68]. If this is the case, then depending on the frequency with which viruses shift between host species, or between individuals within a host species that display substantially divergent immune responses, we might expect selection acting on viruses to be episodic rather than ubiquitous, consistent with our results for VSRs. In the future, it would be very interesting to ask whether the shift between hosts is associated with transiently elevated d*N*/d*S* ratio. However, the extremely wide potential host range of some plant viruses, combined with the relatively poor sampling of viral lineages from non-crop plants and the small samples sizes available, precludes this analysis at present.

## 5. Conclusions

Although VSRs are predicted to be a focus of antagonistic host–virus interaction [5,6], we found little evidence for ubiquitous positive selection acting on the VSRs of plant viruses. Since our analyses are likely to have good power, we believe this is a robust result. However, VSRs do show slightly elevated rates of non-synonymous to synonymous substitution, and this appears to be associated with elevated rates of episodic selection and possibly with broad host range. Given the different time scales of host and pathogen evolution, ubiquitous selection driven by reciprocal arms races will be difficult to detect between viruses and eukaryotes, and therefore our results do not rule out the possibility of arms races having occurred between VSRs and antiviral RNAi genes, but instead demonstrate that these dynamics do not dominate the recent, observable evolution of the virus. The selective forces that we do detect are consistent with the type of selection that could be imposed by frequent shifts between selective environments, such as host shifts or local adaptation to host genotypes.
